# An Impact Assessment Tool to Identify, Quantify and Select Optimal Social-Economic, Ecological and Health Outcomes of Civic Environmental Management Interventions, in Durban South Africa

**DOI:** 10.1016/j.jenvman.2021.113966

**Published:** 2021-11-08

**Keywords:** ecosystem services, environmental management, civic ecology, social-ecological system, sustainable development, environmental impact assessment

## Abstract

Using an environmental impact assessment (EIA) methodology, we provide a novel approach to identify and assess social-ecological outcomes from civic ecology interventions. We quantified the impact significance of six civic (community led) interventions implemented by the Wise Wayz Water Care (WWWC) local community programme (solid waste management, water quality monitoring, invasive alien plant control, crop production, recycling and community engagement), in two communities, situated in urban to peri-urban/rural environments in Durban, South Africa. Interventions resulted in 38 outcomes, of which 37 were positive and one negative. The resulting significance scores from the impact assessment allowed for interventions and their outcomes to be compared. The socio-economic outcomes were the greatest (21), followed by ecological (11) and health outcomes (6). Outcomes included access to education and training; improved quality of life; improved terrestrial and aquatic ecosystems; increase in recreation and cultural uses of natural areas; reduced health risks and increased nutrition. The most significant ecological outcomes resulted from invasive alien plant control, followed by solid waste removal and water quality monitoring. The greatest health outcomes resulted from solid waste removal and vegetable gardens, whereas the greatest social-economic outcomes resulted from the general operation of WWWC, solid waste removal, and invasive alien plant control. We demonstrate that investments in natural areas can deliver not only on enhancements in ecosystems and their services, but also for local community social-economic and health benefits. This study provides an intervention quantifying tool for practitioners to select optimal local management interventions, that can be aligned with desired outcomes related to specific community challenges and policy requirements. In so doing, this work shows the critical role that civic interventions play to ensure sustainability, and emphasises how social-ecological systems and ecosystem services perspectives can be used in practice towards achieving sustainable outcomes.

## Introduction

Human well-being and natural capital are inextricably linked through ecosystem services (Costanza et al. 1997, Daily 1997, MEA 2005, Teeb 2011, Díaz et al. 2018). Biodiversity and ecosystem services (ES) are in rapid decline (IPBES, 2019), placing human well-being at risk (MEA, 2005). The direct drivers of change are from land and sea uses; exploitation of organisms; climate change; pollution; and invasive alien species. All these drivers of change are underpinned by society’s values and behaviours, such as local governance, consumption and production patterns, trade and technological innovations, and population dynamics (IPBES, 2019). Natural capital and ecosystem services are under pressure and at risk due to rapid urbanisation and increasing population growth (Steffen et al., 2015; United Nations, Department of Economic and Social Affairs, 2015) raising the importance of the management and sustainable use of resources, as policy issues (Greenhalgh and Hart, 2015).

Current trajectories of biodiversity and ecosystem services loss may result in global targets, such as the Sustainable Development Goals (SDGs), not being met, with progress being undermined for 80% of the targets related to poverty, hunger, water, health, climate, cities, land and oceans (IPBES, 2019). Radical changes are needed to achieve sustainability, through seeking to facilitate major shifts in understanding and actions, across a range of diverse actors, at both organisational and individual levels (McPhearson et al., 2021).

In Africa, the production of ES declined due to inadequate management (Munang et al., 2011). South Africa has the world’s highest GINI income coefficient of 0.68, with high levels of poverty and inequality (World Bank 2014). In the cities of the South, there is increasing demands for ecosystems and their services (Holden and Otsuka, 2014; Steffen et al., 2015). The growing strain on ecosystem services is concerning for the well-being of more impoverished communities, many of whom are directly dependant on ecosystem services for survival (Shackleton and Shackleton 2004, Stoian 2005, Davenport et al. 2012).

Environmental management is critical to protect biodiversity and ES that support human well-being (Davids et al., 2016). Cities play a crucial role in managing biodiversity and responding to global environmental change issues (Puppim de Oliveira et al., 2011). More locally, in the city of Durban, there are numerous factors limiting the effective management of ES, including that the majority of important ES areas are located outside of formally managed conservation areas, and within jointly administered communal lands, i.e. under joint tribal authority and municipal administration (Davids et al., 2016). These challenges call for alternative solutions and support for the management of natural areas that supply ES, particularly in impoverished communities where ES are being used.

Civic ecology (or ‘local community environmental management’) initiatives could provide opportune alternatives and contribute to more formal environmental management in cities, as they enhance natural capital, ES, and human well-being through environmental stewardship and participatory approaches (Krasny et al., 2013). Civic ecology programmes are recognised for their broad ranging benefits and therefore warrant increased governance support (Davids et al., 2021). Although civic ecology initiatives include collective decision-making as part of adaptive management processes (Jordan et al., 2019), decision-making support for selecting optimal civic ecology interventions is lacking. The challenge remains to measure and assess civic ecology interventions in terms of their contribution to ecological, social and economic outcomes and to provide a tool to facilitate informed decision-making in civic ecology practices. Furthermore, although civic ecology practices are increasing, and contributing to global sustainability initiatives, their contributions to ecosystem services are rarely measured (Krasny et al., 2013).

The objective of this research is to highlight the critical role that civic interventions play to ensure sustainability, and to show how social-ecological systems (Folke et al., 2016; Pacheco-Romero et al., 2020) and ecosystem services (ES) perspectives (Biggs, R., Schlüter, M. and Schoon 2015; Davids et al. 2021) can be used to effectively select- and encourage policy support for- local interventions that will contribute to a multitude of sustainability outcomes and uplift local communities.

Our key questions are: What are the ecological, socio-economic and health outcomes of the community-based environmental management interventions? What is the significance of the impacts of community-based environmental management interventions on the social-ecological system? How can the significance of the ecological, social and health outcomes associated with different local scale civic management interventions be measured in a practical way that is accessible to decision-makers?

In South Africa, Environmental Impact Assessment (EIA) is the key legislated and regulated tool used to ensure ‘integrated environmental management,’ which is a code of practice ensuring that environmental considerations are fully integrated into all stages of the development and decision-making processes at the local, national and international level (Department of Environmental Affairs (DEA), 2014). We used an adapted EIA methodology to quantify and assess the socio-economic, health, and ecological impacts and outcomes from community civic ecology interventions. This was done in order to identify a new quantification tool that can be used as a mechanism for the selection of optimal interventions by practitioners.

**Figure 1:**
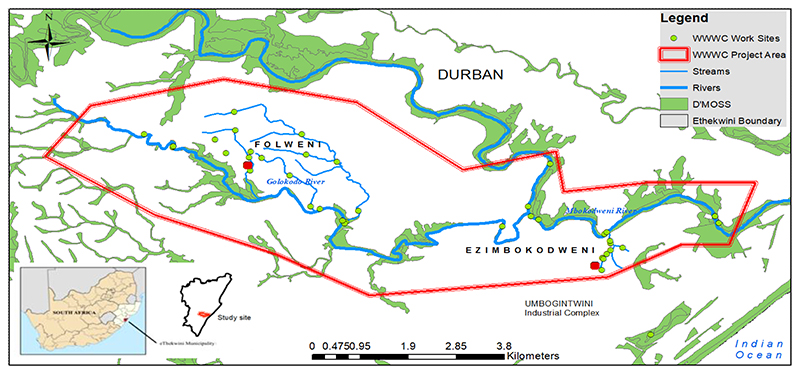
Study area: Wise Wayz Water Care Work Areas in eThekwini Municipality (Durban), South Africa

As a case study, we assessed the interventions of the private sector funded Wise Wayz Water Care (WWWC) programme, being implemented by community members of two low-income peri-urban communities (the beneficiaries), along the Golokodo and Mbokodweni Rivers, within Durban, South Africa (Fig 1).

## Methods

### 1.1.1 Study area

The WWWC work area, the study area (Fig 1), is situated in two rural/peri-urban communities, Folweni (more urban) and Ezimbokodweni (rural-peri-urban), located in Durban, in the province of KwaZulu-Natal, South Africa. The study area is characterised as one of the poorest in Durban, with low education, employment and income levels and low service delivery (Folweni 53% of households have piped water inside the dwelling, 42% have flush toilets connected to sewer, and Ezimbokodweni 10.7% households have piped water inside the dwelling, 4% have a flush toilet connected to sewer) (Department of Statistics South Africa, 2020).

The study site is traversed by the Mbokodweni and Golokodo Rivers and falls within the KwaZulu-Natal Coastal Belt vegetation type within the Indian Ocean Coastal Belt Bioregion (Mucina and Rutherford, 2006), which is classified as endangered. Numerous wetlands are present along the rivers, and the site is traversed by the Durban Metropolitan Open Space System (D’MOSS) (McLean et al., 2016) (Fig 1). D’MOSS is a formal municipal planning policy instrument that identifies a series of interconnected open spaces that incorporate areas of high biodiversity value and natural areas (Davids et al., 2016), with the purpose to protect the globally significant biodiversity (located within the Maputo-Pondoland Biodiversity Hotspot), and ES within the city (Roberts and Donoghue, 2013). See Davids et al. 2021 for more details on the study area.

### 1.1.2 Case study: Wise Wayz Water Care Programme

The Wise Wayz Water Care (WWWC) programme is a civic ecology programme that commenced in 2016, where volunteer groups mainly did litter removal along the Mbokodweni and Golokodo river systems. WWWC was later formalised through funding from the private sector, which facilitated education and training for beneficiaries (community members implementing civic ecology interventions) to implement the six interventions assessed in this study, within the community and in natural areas in and around Ezombokodweni and Folweni.

The six environmental management interventions assessed are (1) Solid waste management and removal: removal of waste from aquatic and terrestrial areas; (2) Recycling: waste collection and storage for recycling; (3) Invasive alien plant control: identification and control of invasive alien plants along rivers and streams; (4) Water quality monitoring: monthly biophysical monitoring of river water quality; (5) Community vegetable gardens: vegetable production (two gardens) using permaculture methods; (6) Community engagement: door-to-door community engagement, surveys, and knowledge sharing. Interventions were implemented at 20 sites within the lower Mbokodweni catchment: within Folweni (11) and Ezomkodweni (9), along various rivers, tributaries, wetlands, and open areas (Fig 1). Interventions assessed in this study were undertaken for three years from 2016 to 2018.

### 1.2 Identification and assessment of social-ecological system impacts, and outcomes of interventions

In order to do the impact assessment, data on the changes to the ecological, socio-economic and health conditions of the community, in response to the interventions, were identified. Data for the environmental impact assessment (EIA) was collected through site visits, stakeholder engagement (surveys and interviews), a social-ecological workshop and specialist study reviews, as described below.

#### 1.2.1 Site visits

Site visits of WWWC work sites were done to identify the general living conditions of the community in the study areas (including housing, water supply, waste management, etc.), and the biophysical condition of the areas where the WWWC interventions were implemented (including wetlands and rivers, open spaces, etc.), through direct field observations and on site discussions with beneficiaries.

#### 1.2.2 Surveys

Three surveys were conducted surveys with beneficiary, community, and external stakeholders (including the WWWC funders: African Explosives and Chemical Industry (AECI) Community Education and Development Trust, government stakeholders: eThekwini Municipality, and the South African National Biodiversity Institute (SANBI) (SM Appendix 1). Surveys were undertaken to identify individual understanding and perceptions of the WWWC programme and associated benefits to the community and beneficiaries, as well as the environment and ES use, and also to gather data on the social, ecological, and economic attributes of the study area (Davids et al., 2021; Nkambule et al., 2016). These surveys also collected socio-economic and health data of participants. Open-ended questions were designed to extract perceptions of the value of the programme to the social–ecological-system of the study area. The three surveys were (1) beneficiaries survey conducted in a workshop setting (N = 60), (2) community survey conducted at randomly selected households along the Mbokodweni and Golokodo rivers (N = 60), and (3) key stakeholder online surveys conducted via Survey Monkey (N = 6). As required by the Ethical Approval, informed consent to utilise the outcomes of the study for research purposes was obtained from all participants. The questionnaires were translated into IsiZulu, allowing participants to choose the language of their preference. Grounded Theory was used to code the data collected in the surveys, whereby the main themes from open ended questions were identified from the data, and not from a preconceived hypothesis (Charmaz, 1996). To ensure intercoder reliability, two of the authors participated in the survey coding, and engaged in discussions to reach consensus regarding the coded responses (Lombard et al., 2005). Data collected via the surveys were analysed using Statistical Package for Social Sciences (SPSS) 25. This study is limited in that surveys were only conducted after interventions were implemented. See Davids et al. 2021 for detailed survey results.

#### 1.2.3 Social-ecological system workshop with beneficiaries

In order to better understand the social-ecological system of the study area, we hosted a workshop with WWWC beneficiaries (n=60), who were randomly selected from the list of beneficiaries. We used A0 size maps as the focus of discussions, which showed the locations of WWWC work areas (WWWC programme boundary and locations of management intervention sites, e.g. water quality monitoring points and solid waste removal sites). Maps were drawn using ArcGIS 10.4, and showed the WWWC work sites relative to other landscape attributes and ecological habitats, namely, the D’MOSS, including wetlands, rivers and vegetation habitats. Beneficiaries reflected on the maps and related their experiences in the study area. Key questions that were explored in the workshop related to existing or perceived understandings of: (1) opportunities related to social activity, knowledge sharing and natural resource use (e.g. water extraction, livestock grazing, and watering); (2) potential expansion of WWWC work areas; and (3) threats relating to health and safety, such as sources of pollution, and illegal dumping of solid waste.

#### 1.2.4 Intervention Impact Assessment

We identified social-ecological outcomes of the WWWC programme from the surveys, workshop and site visits. Outcomes were identified for each of the six interventions, and were categorised into three themes, (1) ecological, where the intervention resulted in the impacts on nature (or ‘natural capital’) (n = 6), (2) socio-economic, where the intervention resulted in the impacts on social or economic aspects (n = 16), and (3) health, where the intervention resulted in impacts on health (n = 3). We then scored the impact significance, either positive (+) or negative (-), for each outcome.

To quantify the significance of the outcomes of the WWWC interventions, we used an adapted EIA method, based on the general approach to impact significance assessment applied in South Africa (Department of Environmental Affairs and Tourism, 2002), and the requirements for impact assessment in the 2017 Amendments of the Environmental Impact Assessment Regulations, 2014 (DEA GNR 326, 2017). Innovation in impact assessment theory and practice has occurred over time, and has resulted in new concepts aimed at integrating biophysical and socio-economic data (Costanzo and Sánchez, 2019), including the incorporation of ecosystem services (Rosa and Sánchez, 2016). The innovation in the methods applied here relates to the application of EIA on civic ecology practices. We believed Environmental Assessment was a suitable approach, potentially the most successful environmental policy intervention of our time, that is used internationally (Taylor et al., 2012), and which allows for more sustainable outcomes to be achieved (Sandham and Retief 2016), through the quantification of ecological, socio-economic, and health impacts. Furthermore, impact assessment that incorporates consideration of environmental and social impacts using an ecosystem service approach has been shown to improve EIA practice (Rosa and Sánchez, 2016). The lead Author is also a Registered Environmental Assessment Practitioner (EAP) under the Environmental Assessment Practitioners Association of South Africa (EAPASA) and drew on her experience undertaking EIAs in South Africa.

Outcomes were ranked and scored in terms of five assessment criteria (Department of Environmental Affairs and Tourism, 2002): (1) Extent: spatial scale of the impact; (2) Magnitude: degree of the impact; (3) Duration: time scale of the impact; 4) Reversibility: degree to which the outcome can be reversed; and 5) Probability: of the impact occurrence (SM Table 1). The manner of identifying the rank of each of the criteria (Table 1) is based on the EAP’s professional judgement and knowledge of the site and activities as gathered through data collection. Using these five assessment criteria, the significance of each outcome was determined, whereby the significance (**S**) of the impact is determined by the probability (**P**) of the particular impact occurring, and the consequence (**C**) of the impact. The consequence is determined by combining the spatial (geographical) extent (**E**), magnitude (**M**), duration (**D**), and reversibility (**R**), applicable to the specific impact using the formula below (Table 1) (Department of Environmental Affairs and Tourism, 2002). Positive impacts resulted in (+) scores, while negative impacts, resulted in (-) scores. Thus, negative impacts would reduce overall impact significance scores, when totalled with positive scores.

S=C(E+M+D+R)/4)×P.

Outcomes were scored and classified into three categories of impact significance (high, moderate, or low (Table 2). Interventions were also scored to assess their relative cumulative impact across the three broad categories of ecological, socio-economic, and health, whereby high impact = 10, moderate = 5 and low = 3. This was done to compare interventions in terms of outcome categories (SM Table 2).

The assessment considered outcomes related to the six WWWC interventions, and the general operation of the programme. While the sixth intervention, community engagement, does not involve practical management, it provides a critical component that facilitates the achievement of environmental management actions through community education and indirectly results in social-ecological improvements. Similarly, the ‘general operation of WWWC programme’ was also assessed, to consider those impacts that fell outside of specific interventions.

#### 1.2.5 Verification of ecological impacts: review of specialist studies

To verify ecological impacts of the project for the impact assessment, we used two specialist studies commissioned by the private sector funder, AECI, to monitor the impact of the WWWC interventions on the aquatic environment within the study area (GroundTruth 2016, 2017). The first study was a baseline assessment of Golokodo and Mbokodweni Rivers conducted in 2016 (wet and dry season sampling, i.e. August and November respectively), which included biological and chemical assessments of both rivers namely, assessment of present ecological state; South African Scoring Systems (SASS5); bethnic diatoms (algea); riparian health audit, and physico-chemical water quality assessments. This baseline assessment was used as a benchmark in 2017 wet and dry seasons to measure change after WWWC interventions had been implemented. River health was classified into five classes, from natural, good, fair, poor, and being seriously modified.

#### 1.2.6 Identifying natural capital and ecosystem services in the system

Natural resources included in the definition of ‘natural capital’ are locally available and are directly and regularly used by households (e.g. freshwater from a natural source, fuelwood, rangeland for grazing livestock) (Hamann et al., 2015; Reynolds et al., 2020). NC provisioning areas in the study area were classified according to spatial information contained in the D’MOSS (Fig. 1), namely, the Mbokodweni and Golokodo rivers, wetlands, forest patches, woodlands, thickets, and grassland habitats. ES were identified from survey responses, based on the existing use or demand for that service by community members and beneficiaries. Surveys (as described above) were used to collect data on ES usage by (access), and values of ES. The ES included in the survey were (1) River water use: use of natural water from river or stream (e.g. for washing clothes and cars or for general household use; (2) Natural material harvesting: gathering natural materials for various uses, e.g. medicinal plants and wood; (3) Subsistence use: direct use of natural resources to sustain life, e.g. food and water (4) Agricultural use: crop or livestock production; (5) Cultural practices: use of natural areas for cultural practices or rituals; and (6) Recreation and leisure: use of natural areas for leisure and outdoor activities. See Davids et al. 2021 for detailed outcome of the ES survey. Additionally, ES expected to be enhanced by the civic management interventions were identified based on literature (Díaz et al. 2018; de Groot et al. 2010), relative to the type of ecological habitat affected, or mitigated habitat impacts, and ecosystem functions (Table 3 and SM Table 1).

#### 1.2.7 Identifying strengths linkages between interventions and outcomes

We used bipartite analysis to describe the pattern, and visualise the strengths of linkages, between the interventions and the identified outcomes. The analysis was done in R using the package "bipartite" (Devoto et al., 2021). This visualisation allowed for the cumulative effects of each intervention, based on the impact assessment scores, to be shown.

## Results

### 1.3 Impact Assessment of Civic Interventions

**Figure 2:**
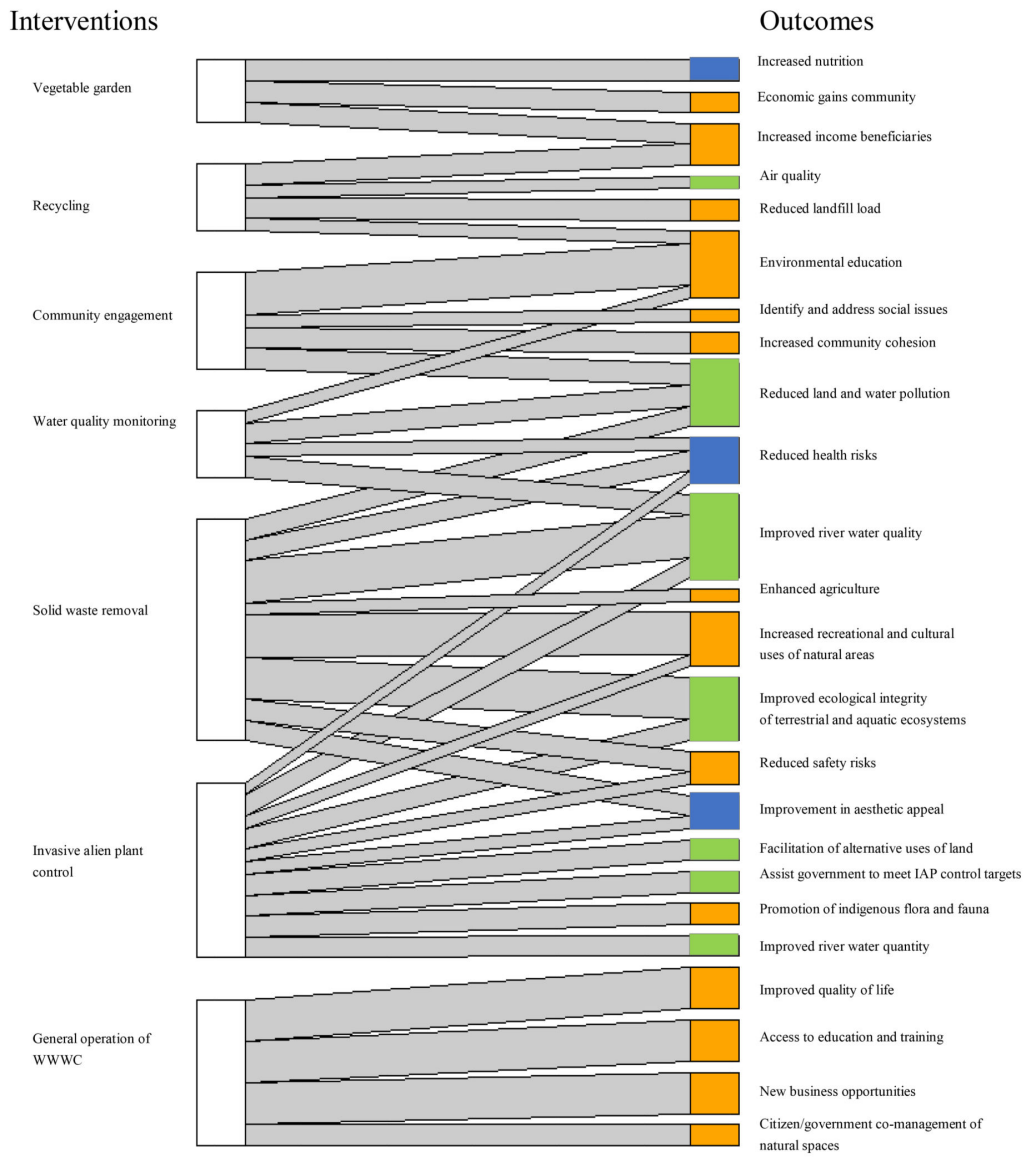
Impact significance of intervention outcomes: Here, we show the interrelationships between the interventions (on the left) and all the resulting social (orange), ecological (green) and health (blue) impacts/ human well-being outcomes (on the right). The width of bars denote the significance scores as calculated in the impact assessment (the higher the score, the thicker the line). Significance scores were calculated using an Environmental Impact Assessment methodology, whereby the significance (S) of the impact was determined by the probability (P) of the impact occurring, times the consequence (C) of the impact. The consequence is determined by combining the extent (E), magnitude (M), duration (D), and reversibility (R) of the impact). IAP has an extra arrow in this diagram compared to the number of impacts in the assessment, where ‘improvement of 1) water quality and 2) water quantity’ were combined (now they are listed as two separate outcomes).

We identified and assessed the impact significance of 38 outcomes in total, from the six civic interventions (SM Table 1), of which, the socio-economic outcomes were the greatest (n=21), followed by ecological (11), and health outcomes (6). The impact significance of the outcomes for each individual intervention was identified (Fig. 2, SM Table 1, SM Appendix 2). For ease of illustration, Table 3 shows the assessment for ‘solid waste removal’ (SM Table 1 contains the assessment results for all interventions)

All the interventions assessed were found to have positive impacts on the beneficiaries, the broader community, and their natural surroundings, including both aquatic and terrestrial environments. Only one negative impact was identified from general operation of WWWC, namely, that the programme caused ‘conflict in the community’ related to some beneficiaries having received cost recovery (provided to beneficiaries for carrying out activities in addition to basic activities), while others did not (SM Table 1). Invasive alien plant control and solid waste removal had the most positive outcomes (eight each), followed by community engagement and general operation of WWWC (five outcomes) (Fig. 2).

Significance scores were ranked as high, medium or low and ranged from a score of 4,5 (low positive impact) to 22,5 (high positive impact). The two outcomes that had the highest significance scores (22,5 and 21,25) were from the general operation of WWWC, namely, access to education and training of beneficiaries and improved quality of life of beneficiaries and community members (denoted by the thicker links in Fig. 2). This was followed by an increase in recreation and cultural uses of natural areas, from the solid waste removal intervention, with a significance rating of 18,75.

### 1.4 Nature and ecosystem services enhanced

The natural areas that were enhanced by the interventions include terrestrial and aquatic habitats, e.g. wetlands, rivers/streams, riparian vegetation, and open space (natural areas zoned as public open space). The interventions positively impacted ecological areas and were, thus, considered to have the potential to enhance ES (SM Table 1). The habitats improved by the interventions are linked to the enhancement of numerous ES (Davids et al., 2021), including regulating services or Nature’s Contributions to People (NCP), of water purification, flood mitigation, biological regulation, and/or disease control, and maintenance of biological diversity (genepool protection) (previously considered a supporting service MEA, 2005), but now captured in regulating NCP (Díaz et al. 2018); cultural or non-material NCP of aesthetic, recreational, cultural, and education service; provisioning services or material NCP of water supply, food, and harvesting products (Díaz et al. 2018; de Groot et al. 2010). People accessed ES for water, agricultural production, and harvesting of medicinal plants and wood (SM Table 1), and increased use of natural spaces for cultural and spiritual activities since it had been cleaned by the beneficiaries, for example, using the wetland in Ezimbokodweni for cultural rituals (*Umemelo* - Zulu traditional coming of age ceremony for women) (Davids et al., 2021).

### 1.5 Comparison of impact categories per intervention

**Figure 3:**
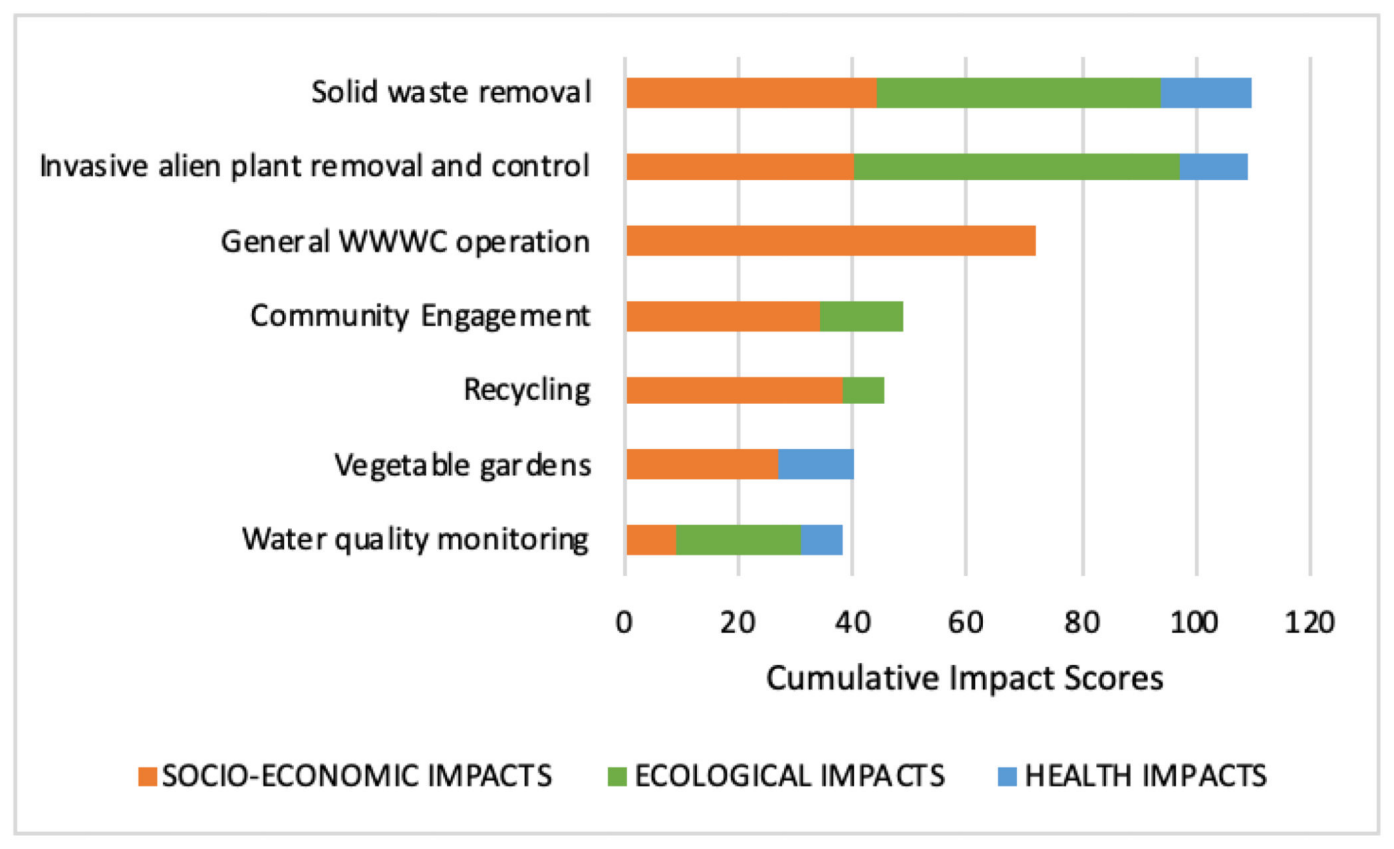
Cumulative impact significance scores as calculated for each community intervention, relative to three categories of outcomes, i.e., socio-economic, ecological and health.

WWWC interventions resulted in a combination of socio-economic, ecological, and health outcomes. Outcomes resulted in an enhancement of the social-ecological system as a whole, and were able to address a multitude of community issues. When comparing the outcomes of interventions per category (Fig. 3, SM Table 2), the interventions that resulted in the impacts of the greatest ecological significance were invasive alien plant control (score of 57 from four outcomes), followed by solid waste removal (score of 50 from three outcomes), and water quality monitoring (score of 22 from two outcomes). Solid waste removal and vegetable gardens resulted in the greatest health outcomes (scores of 16 from 3, and 13 from one outcome, respectively), whereas general operation of WWWC, solid waste removal, and invasive alien plant control, resulted in the greatest socio-economic outcomes. Overall, solid waste removal scored highest in terms of cumulative impacts combined for all categories, followed very closely by invasive alien plant control.

In most cases, a particular positive outcome resulted from multiple interventions (Fig. 2, SM Table 2). Examples of these include: (1) ‘Improved river water quality’ and ‘reduction in health risks; resulted from three interventions, namely, solid waste removal, invasive alien plant control, and water quality monitoring; (2) ‘Improved ecological integrity of terrestrial and aquatic systems’ resulted from solid waste removal and invasive alien plant control; 3) ‘Reduced safety risks’, ‘improvement in aesthetic appeal’, and ‘increase in recreational and cultural uses of land’, resulted from solid waste removal and invasive alien plant control. Thus, the interventions resulted in cumulative impacts that together increased the significance of those positive outcomes.

## Discussion

In line with recent research, this study confirms that the application of the ecosystem service approach in impact assessment is an innovation that improves the assessment of environmental and social impacts, both in the participation of affected communities and the integration of knowledge areas (Rosa and Sánchez, 2016). Although conventional impact assessment is focussed on mitigating negative impacts, our use of EIA on civic ecology initiatives aligns with the call for increased focus on the benefits or enhancements of projects, plans and programmes that facilitate sustainability (João et al., 2011). This research makes three important contributions to the science on civic ecology: (1) We confirm that civic ecology interventions can result in improvements in human well-being through numerous socio-economic, health and ecological enhancements; (2) We provide a novel tool for assessing civic ecology interventions that can assist in selection of optimal interventions and adaptive management; and (3) We share learnings from civic ecology interventions to aid duplication in similar contexts.

### By considering the ‘whole system,’ civic ecology contributions to human well-being can be identified

Our study confirms that civic interventions are important in the protection and management of natural areas that produce ES, while, at the same time, constitute social-ecological processes that enhance a multitude of ecosystem services and human well-being (Ezebilo and Mattsson 2010, Krasny et al. 2013, Díaz et al. 2018). Although most of the interventions were targeted for environmental management, we demonstrate that investments in natural areas can deliver enhancements in ecosystems and their services and social-economic and health benefits (Krasny et al., 2013). Thereby, ultimately contributing to all three foundational components of sustainability and responding to the challenges of the global goals not being met due to biodiversity and ES decline (IPBES, 2019). Furthermore, by showcasing the sustainability of civic ecology, this research calls for support from the government towards local actions that are needed to ensure sustainability (Dearing et al., 2014).

The demonstration of the range of social, ecological, economic and health outcomes (cumulative impacts and bipartite analysis) resulting from management interventions provides further motivation for the selection of interventions for implementation – as the broader the range of positive impacts resulting from the intervention, the greater the potential for the intervention to respond to the wide-ranging issues that government actors face. This, therefore, also forms the basis from which to motivate for political action, given that ecosystem-based approaches provide wins for people and the ecosystems on which they depend (Roberts et al., 2012). Such interventions are particularly important as they enhance the protection and management of ecosystems and their services, as a means to safeguard the livelihoods of citizens, particularly the urban poor (MEA, 2005; Tallis et al., 2008).

Another aspect of this work is that it can inform policy towards achieving national and international goals and targets. Our study provides evidence that civic ecology can enhance natural resources and ecosystems, which play important roles in food production; and have influences on food security, nutrition and health. For example, food security is on both the South Africa Development Agenda (National Planning Commission, 2011) and the global agenda for Sustainable Development (SDG 2) (United Nations, 2015). This study shows that local management of natural systems plays a role in supporting local agriculture, a key feature of food systems that can only be sustainable if natural resources, such as water, soil, and land, are managed appropriately (HLPE, 2016, 2015). The vegetable gardens implemented by the beneficiaries were grown using natural river water and were shown to result in socio-economic and health outcomes. The provision of education and skills to community members also had a positive impact on socio-economic aspects of the community, which has been shown to influence food security (HLPE, 2017). These local actions towards enhancing food security can thus be counted towards national (National Development of South Africa) and global (SDG) targets.

### A novel method for assessing contributions of civic ecology interventions and selecting optimal interventions

This study contributed to the science of measuring civic ecology, which was last proposed by Krasny et al. (2013), by providing a novel method of assessing the contributions of civic ecology interventions. Our application of an environmental assessment methodology to quantify impacts of civic ecology interventions provides a new tool for government practitioners and business funders to select interventions for maximum impact related to desired outcomes (for example, Fig. 2). Our study also contributes to the literature by providing a tool for comparing the significance of civic ecology interventions, which can be useful to determine which interventions to select, particularly when budgets are limited. This allows for sources of improvement (i.e. investments) to be compartmentalised and for impacts of investments to be quantified in an auditable manner. Thereby enhancing both an understanding of return on investment, but also providing for government at various levels (local, provincial, national) to account across sectors. For example, this tool could be useful to assess existing activities or determine the most suitable actions to incorporate into community-focused river rehabilitation programmes part of city management planning (C40 Finance Facility, 2019).

Furthermore, the scoring of intervention impacts can assist the government, and other implementing agents, to identify and select interventions that will have the most significant impacts in response to specific community challenges and policy requirements. For example, one of the most significant social outcomes was increased education of the community on environmental issues (high positive significance). Thus, other practitioners wishing to achieve similar outcomes may wish to incorporate community engagement as an intervention. The civic ecology interventions, such as those considered in this study, could be initiated by local governments who are mandated to ensure service delivery, for example, job creation, supply of water and sanitation and management of natural resources. Such initiatives should then be jointly managed and implemented in partnerships with private actors or community organizations (Castán Broto and Bulkeley, 2013).

### Learnings from civic ecology interventions

This study provided a number of unique reflections on civic ecology initiatives: (1) The financial support received from private industry was instrumental for the success of the WWWC programme. Although the community originally started with basic activities such as solid waste clearing voluntarily, the result of 37 positive outcomes, as identified in this study, relied on the education and skills training that beneficiaries received. For example, the clearing of invasive alien plants was only possible with the knowledge (education) of the legislation guiding the requirements for their removal, which plants needed to be removed, tools for their removal and ecologically accepted removal methods. Majority of which require funding to be achieved. (2) The beneficiaries received stipends for the work they did. Although small in amount, this stipend covered some basic needs of beneficiaries (with some noting they were able to buy food therefrom). As such, the programme was a means of livelihood for many beneficiaries. (3) The funding was managed by an implementing agent, who worked closely with the community members and beneficiaries to identify the specific needs of the community (in relation to challenges they were facing) and then, sourced the required training, skills and equipment, and assisted with the drafting of a schedule of activities/tasks that the beneficiaries implemented, in response to the community’s needs. For duplication of the interventions discussed in this study, sourcing funding would be crucial. Furthermore, time is needed to understand specific challenges faced in a particular community, to allow for adequate planning of responses through the civic ecology interventions.

A limitation of this study is that the financial costs of implementation of each intervention were not factored in. This study could be expanded by taking into account the costs of training needs, the number of people workdays and equipment needed, which would provide an additional factor in determining the optimal interventions to be selected by practitioners. Additionally, the monetary quantification of benefits from each intervention, not done here, could increase understanding of cost vs benefits in selecting interventions. Such interventions should aim to incorporate environmental, social, and economic dimensions, including the sustainable use of ecosystem goods and services, promoting dignified standards of life, and providing employment opportunities (Ezebilo and Mattsson, 2010).

The lead Author holds the professional requirements for undertaking EIAs and could therefore implement the methods used here, based on the ethical codes of conduct and practice for environmental assessment practitioners in South Africa (EAPASA, 2016). The duplication of the methodology by non-practitioners should take cognisance of potential errors that could occur related to the assessment of impacts. For example, errors in the identification and scoring of impacts could result if they were not informed by specialist studies where needed (as done for EIAs), or if not undertaken in an interdisciplinary manner through collaboration and engagement with local communities and key stakeholders (EAPASA, 2016).

Despite these limitations, this work can be utilised as a tool for the identification, selection, monitoring and adaptive management of civic ecology programmes elsewhere. Thus, ensuring that investments in natural areas that deliver optimal enhancements in ecosystems and their services, as well as local community social-economic and health benefits.

## Conclusion

This work shows the critical role that civic interventions play to ensure sustainability, and emphasises how social-ecological systems and ecosystem services perspectives can be used in practice towards achieving sustainable outcomes. By using the ES concept, we emphasised the importance of the ‘whole system’ and not just humans (Costanza et al., 2017). Our use of EIA methodology for assessing the impact of civic interventions can be easily duplicated, and thus provides an additional tool to assist decision-makers and funders in the selection of interventions that can result in improved social-ecological system outcomes. The multiple benefits of improving the environment while also achieving health improvements and social upliftment is a model that can be duplicated in other parts of the world with similar social-ecological conditions. We provide, test, and learn from a conceptual framing that is holistic and systematic and demonstrate the opportunities that can be brought about through mutually beneficial relationships between humans and the environment at the local community level, but which could be effectively upscaled for broader societal impact.

## Supplementary Material

Supplementary Material

## Figures and Tables

**Table 1:** Evaluation and ranking criteria to assess the impact significance of intervention outcomes (based on Department of Environmental Affairs and Tourism, 2002)

Evaluation components	Ranking scale and description criteria
MAGNITUDE of NEGATIVE IMPACT (at the indicated spatial scale)	5 - Very high: Bio-physical and/or social functions and/or processes might be severely altered. 4 - High: Bio-physical and/or social functions and/or processes might be considerably altered. 3 - Medium: Bio-physical and/or social functions and/or processes might be notably altered. 2 - Low : Bio-physical and/or social functions and/or processes might be slightly altered. 1 - Very Low: Bio-physical and/or social functions and/or processes might be negligibly altered. 0 - Zero: Bio-physical and/or social functions and/or processes will remain unaltered
MAGNITUDE of POSITIVE IMPACT (at the indicated spatial scale)	5 - Very high (positive): Bio-physical (air, water, soil, wetlands) and/or social (human wellbeing) functions and/or processes might be substantially enhanced. 4 - High (positive): Bio-physical (air, water, soil, wetlands) and/or social (human wellbeing) functions and/or processes might be considerably enhanced. 3 - Medium (positive): Bio-physical and/or social (human wellbeing) functions and/or processes might be notably enhanced. 2 - Low (positive): Bio-physical and/or social (human wellbeing) functions and/or processes might be slightly enhanced. 1 - Very Low (positive): Bio-physical and/or social (human wellbeing) functions and/or processes might be negligibly enhanced. 0 - Zero (positive): Bio-physical and/or social (human wellbeing) functions and/or processes will remain unaltered.
DURATION (timeframe during which the impact will be experienced	5 - Permanent 4 - Long term: > 10 years or until the activity ceases. 3 - Medium term: 1- 10 years 2 - Short term: < 1 year. 1 - Immediate
EXTENT (spatial scale/influence of impact)	5 - International: Beyond National boundaries. 4 - National: Beyond Provincial boundaries and within National boundaries. 3 - Regional: Beyond 5 km of the proposed development and within Provincial boundaries. 2 - Local: Within 5 km of the proposed development. 1 - Site-specific: On site or within 100 m of the site boundary. 0 – No impact
REVERSIBILITY of impact (can the impact of the intervention be reversed?)	5 – Impact cannot be reversed. 4 – Low potential that impact might be reversed. 3 – Moderate potential that impact might be reversed. 2 – High potential that impact might be reversed. 1 – Impact will be reversible. 0 – No impact.
PROBABILITY (of occurrence). In most cases, the impact has occurred as the intervention has been implemented. Thus, many impacts score 5 in this category.)	5 - Definite: The impact will occur. 4 - High probability: It is most likely that the impact will occur (>75% chance) 3 - Medium probability: the impact may occur (50% - 75% chance) 2 - Low probability: 25% - 50% chance that the impact may occur. 1 - Improbable: <25% chance of the potential impact occurring.
CUMULATIVE Impacts	High: The activity is one of several similar past, present or future activities in the same geographical area, and might contribute to a very significant combined impact on the natural, cultural, and/or socio-economic resources of local, regional or national concern. Medium: The activity is one of a few similar past, present or future activities in the same geographical area, and might have a combined impact of moderate significance on the natural, cultural, and/or socio-economic resources of local, regional or national concern. Low: The activity is localised and might have a negligible cumulative impact. None: No cumulative impact on the environment.

**Table 2:** Rating scale for intervention outcomes

Significance Score	Significance	Description
≥ 17	High	This impact will affect ecological, socio-economic and health functions and will result in a significant benefit or risk.
≥ 10 < 17	Moderate	The impact is of medium significance may have an effect on ecological, socio-economic and health functions, and could result in a moderate benefit or risk.
< 10	Low	The impact of low significance is not likely to affect the ecological, socio-economic and health functions in a noticeable way and is unlikely to result in significant benefit or risk.

**Table 3:** Impact Assessment of interventions contributions and linkages to natural capital, ES and community issues responded (solid waste removal shown here as an example of the methodology, See SM Table 1 for all other calculations)

WWWC Intervention	Category of impact	Outcomes	Magnitude	Duration	Extent	Reversibility	Probability	Total Impact Significance Points	Significance rating (+/-)	Natural capital affected	Ecosystem function	Ecosystem services enhanced	Community issues responded to
								(Total) × P					
**Solid waste removal** This activity results in the reduction of waste on land and in water courses.	**Ecological**	Improved river water quality.	4	4	2	4	5	17,5	High (+)	Riparian vegetation, rivers, streams, wetlands		Water purification	Solid waste pollution
		Improved ecological integrity of terrestrial and aquatic ecosystems.	4	4	2	4	5	17,5	High (+)	Terrestrial and aquatic habitats, e.g. wetlands, rivers/streams, open space	Water flow regulation Maintenance of ecological balance	Flood mitigation Hazard mitigation Maintenance of biological diversity (genepool protection)	Poor waste collection service delivery
		Reduction in land and water pollution.	5	4	1	2	5	15	Moderate (+)	Riparian plants and soil	Biotic and abiotic processes in breakdown of organic matter, nutrients and compounds	Waste assimilation	Solid waste pollution
	**Socio-economic**	Reduced safety risks to animals and children.	3	4	1	4	4	12	Moderate (+)			Recreational service	
		Improvement in aesthetic appeal of the area.	4	4	1	4	5	16,25	Moderate (+)	Terrestrial and aquatic habitats, e.g. wetlands, rivers/streams, open space	Aesthetic quality of natural area	Aesthetic service	Injury to animals and children from solid waste pollution Unsightly pollution dumps
		Increase in recreational and cultural uses of natural areas.	5	4	2	4	5	18,75	High (+)	Terrestrial and aquatic habitats, e.g. wetlands, rivers/streams, open space	Presence of natural features	Recreational service Cultural service	Lack of recreational space due to pollution
		Enhancement of agriculture due to improved water quality	3	4	1	4	3	9	Low (+)	Aquatic habitats, e.g. wetlands, rivers/streams	Water supply and purification for irrigation Soil retention by vegetation preventing loss of topsoil	Agricultural service Erosion control	Food insecurity
	**Health**	Reduction in health risks related to diseases linked to pollution, e.g. skin rashes, cholera.	4	4	1	4	4	13	Moderate (+)	Aquatic habitats, e.g. wetlands, rivers/streams	Control of pest populations	Biological regulation/disease control	Water-borne diseases
