# Supplementary material for: An Impact Assessment Tool to Identify, Quantify and Select Optimal Social-Economic, Ecological and Health Outcomes of Civic Environmental Management Interventions, in Durban South Africa
Source: J Environ Manage. Author manuscript; Available in PMC 2023 Sep 5. (PMC7615018; doi:10.1016/j.jenvman.2021.113966)
Supplement: Supplementary Material [file EMS186813-supplement-Supplementary_Material.docx]

**SUPPLEMENTARY MATERIAL**

**SM Tables**

SM Table 1: Intervention Impact Assessment Scoring Table

SM Table 2: Intervention Categories Cumulative Impact Scoring

**SM Appendices**

SM Appendix 1: Questionnaires

SM Appendix 2: Descriptions of Intervention Impact Significance

SM Table 1: Intervention impact Assessment Scoring Table

| **WWWC Intervention** | **Category of impact** | **No.** | **Outcomes** | **Magnitude** | **Duration** | **Extent** | **Reversibility** | **Probability** | **Total Impact Significance Points** | **Significance rating (+/-)** | **Natural capital affected** | **Ecosystem function** | **Ecosystem services enhanced** | **Community issues responded to** |
| --- | --- | --- | --- | --- | --- | --- | --- | --- | --- | --- | --- | --- | --- | --- |
|  |  |  |  |  |  |  |  |  | **(Total) x P** |  |  |  |  |  |
| **Solid waste removal**  This activity results in the reduction of waste on land and in water courses. | **Ecological** | **1** | 1. Improved river water quality. | 4 | 4 | 2 | 4 | 5 | 17,5 | High (+) | Riparian vegetation, rivers, streams, wetlands |  | Water purification | Solid waste pollution |
|  |  | **2** | 2. Improved ecological integrity of terrestrial and aquatic ecosystems. | 4 | 4 | 2 | 4 | 5 | 17,5 | High (+) | Terrestrial and aquatic habitats, e.g. wetlands, rivers/streams, open space | Water flow regulation Maintenance of ecological balance | Flood mitigation  Hazard mitigation Maintenance of biological diversity (genepool protection) | Poor waste collection service delivery |
|  |  | **3** | 3. Reduction in land and water pollution. | 5 | 4 | 1 | 2 | 5 | 15 | Moderate (+) | Riparian plants and soil | Biotic and abiotic processes in breakdown of organic matter, nutrients and compounds | Waste assimilation | Solid waste pollution |
|  | **Socio-economic** | **4** | 1. Reduced safety risks to animals and children. | 3 | 4 | 1 | 4 | 4 | 12 | Moderate (+) |  |  | Recreational service |  |
|  |  | **5** | 2. Improvement in aesthetic appeal of the area. | 4 | 4 | 1 | 4 | 5 | 16,25 | Moderate (+) | Terrestrial and aquatic habitats, e.g. wetlands, rivers/streams, open space | Aesthetic quality of natural area | Aesthetic service | Injury to animals and children from solid waste pollution  Unsightly pollution dumps |
|  |  | **6** | 3. Increase in recreational and cultural uses of natural areas. | 5 | 4 | 2 | 4 | 5 | 18,75 | High (+) | Terrestrial and aquatic habitats, e.g. wetlands, rivers/streams, open space | Presence of natural features | Recreational service Cultural service | Lack of recreational space due to pollution |
|  |  | **7** | 4. Enhancement of agriculture due to improved water quality | 3 | 4 | 1 | 4 | 3 | 9 | Low (+) | Aquatic habitats, e.g. wetlands, rivers/streams | Water supply and purification for irrigation Soil retention by vegetation preventing loss of topsoil | Agricultural service Erosion control | Food insecurity |
|  | **Health** | **8** | 1. Reduction in health risks related to diseases linked to pollution, e.g. skin rashes, cholera. | 4 | 4 | 1 | 4 | 4 | 13 | Moderate (+) | Aquatic habitats, e.g. wetlands, rivers/streams | Control of pest populations | Biological regulation/disease control | Water-borne diseases |
| **Recycling**  Impacts form recycling are indirect and result from removal of solid waste. | **Ecological** | **9** | 1. Reduction in air pollution due to avoided burning of waste. | 3 | 4 | 3 | 4 | 2 | 7 | Low (+) | Terrestrial and aquatic habitats, e.g. wetlands, rivers/streams, open space | Capacity of ecosystems to extract pollutants e.g. vegetation / leaf cover | Air purification | Burning of waste due to poor of waste collection service |
|  | **Socio-economic** | **10** | 1. Source of income to recyclers. | 1 | 4 | 1 | 5 | 5 | 13,75 | Moderate (+) |  |  |  | Poverty and low employment |
|  |  | **11** | 2. Provides environmental education to community members on recycling. | 2 | 4 | 1 | 4 | 3 | 8,25 | Low (+) |  |  | Environmental education | Low education |
|  |  | **12** | 3. Reduced load on local landfill and waste removal services. | 2 | 4 | 2 | 5 | 5 | 16,25 | Moderate (+) |  |  | Waste assimilation |  |
| **Invasive alien plant removal and control**  This activity controls the spread of invasive alien plants. | **Ecological** | **13** | 1. Promotion of indigenous species of flora and fauna. | 4 | 4 | 3 | 3 | 4 | 14 | Moderate (+) | Terrestrial and aquatic habitats, e.g. wetlands, rivers/streams, open space | Maintenance of ecological balance | Maintenance of biological diversity (genepool protection) Pollination  Harvesting products | Infestation of invasive alien plants Lack of access to resources |
|  |  | **14** | 2. Improved water quality and quantity. | 4 | 3 | 3 | 3 | 5 | 16,25 | Moderate (+) | Aquatic habitats, e.g. wetlands, rivers/streams | Water flow regulation | Water supply Water purification | Loss of water from rivers due to IAPs |
|  |  | **15** | 3. Improvement in ecological integrity of natural spaces, particularly wetlands and improvement of flow of water and mitigation of flooding. | 4 | 3 | 3 | 4 | 3 | 10,5 | Moderate (+) | Aquatic habitats, e.g. wetlands, rivers/streams | Water flow regulation | Flood mitigation | Loss of water from rivers due to IAPs |
|  | **Socio-economic** | **16** | 4. Facilitation of alternative and more appropriate uses of land, e.g. more land available for community gardens. | 4 | 3 | 1 | 3 | 5 | 13,75 | Moderate (+) | Terrestrial and aquatic habitats, e.g. wetlands, rivers/streams, open space | Water supply and purification for irrigation Soil retention Soil formation | Agricultural service Erosion control | Loss of land due to encroachment by IAPs |
|  |  | **17** | 5. Improvement in aesthetic appeal of the area. | 3 | 3 | 1 | 3 | 3 | 7,5 | Low (+) | Terrestrial and aquatic habitats, e.g. wetlands, rivers/streams, open space | Aesthetic quality of natural area | Aesthetic service | Unsightly overgrown areas. |
|  |  | **18** | 6. Increase in recreational and cultural uses of natural areas. | 3 | 3 | 1 | 4 | 5 | 13,75 | Moderate (+) | Terrestrial and aquatic habitats, e.g. wetlands, rivers/streams, open space | Presence of natural features | Recreational service Cultural service | Loss of land due to encroachment by IAPs |
|  |  | **19** | 7. Reduced safety risks (related to crime) | 3 | 4 | 1 | 4 | 3 | 9 | Low (+) | Terrestrial and aquatic habitats. |  |  | Use of IAP infested areas for criminal activity |
|  |  | **20** | 8. Assist municipality to achieve IAP control targets. | 2 | 3 | 1 | 4 | 2 | 5 | Low (+) | Terrestrial and aquatic habitats, e.g. wetlands, rivers/streams, open space | Maintenance of ecological balance | Maintenance of biological diversity (genepool protection) | No municipal IAP programme in the study area |
|  | **Health** | **21** | 9. Reduction in health risks associated with invasive plants, e.g. through removal of poisonous plants. | 2 | 3 | 1 | 3 | 2 | 4,5 | Low (+) | Terrestrial and aquatic habitats, e.g. wetlands, rivers/streams, open space |  |  | Skin rashes due to contact with certain IAPs |
| **Water quality monitoring** | **Ecological** | **22** | 1. Improved river water quality | 3 | 2 | 2 | 4 | 4 | 11 | Moderate (+) | Aquatic habitats, e.g. wetlands, rivers/streams | Control of pest populations water filtration by wetlands | Water purification | Pollution of rivers due to littering and dumping |
|  |  | **23** | 2. Reduction in land and water pollution. | 3 | 4 | 1 | 3 | 4 | 11 | Moderate (+) | Terrestrial and aquatic habitats, e.g. wetlands, rivers/streams, open space | Biotic and abiotic processes in breakdown of organic matter, nutrients and compounds | Water purification |  |
|  | **Socio-economic** | **24** | 3. Environmental education of community (Empowering community to understand the natural environment and creating awareness of water pollution). | 4 | 3 | 1 | 4 | 3 | 9 | Low (+) | Aquatic habitats, e.g. wetlands, rivers/streams | Presence of natural features of educational value | Educational service | Low education |
|  | **Health** | **25** | 4. Reduced health risks of water borne diseases. | 4 | 1 | 1 | 4 | 3 | 7,5 | Low (+) | Aquatic habitats, e.g. wetlands, rivers/streams | Control of detrimental organisms/ecological processes, e.g. pest and pathogens | Biological regulation/disease control | Water-borne diseases |
| **WWWC vegetable gardens**  Two vegetable gardens are part of the programme that grow a variety of vegetable, fruit and herbs, which are consumed by beneficiaries and sold to the local community and local grocery store. | **Socio-economic** | **26** | 1. Increased income for beneficiaries e.g. though sale of vegetables. | 2 | 4 | 1 | 5 | 5 | 15 | Moderate (+) | Terrestrial and aquatic habitats, e.g. wetlands, rivers/streams, open space | Water supply and purification for irrigation Soil retention by vegetation preventing loss of topsoil | Agricultural service Erosion control Pollination  Harvesting products | Poverty and low employment |
|  |  | **27** | 2. Positive economic impact on community members due to availability of cost effective fresh vegetables locally and reduction in need to travel to purchase vegetables. | 3 | 4 | 1 | 4 | 4 | 12 | Moderate (+) |  |  |  |  |
|  | **Health** | **28** | 3. Health (nutritional) benefits to community through increased accessibility and consumption of a large variety of nutrient rich vegetables. | 4 | 4 | 1 | 4 | 4 | 13 | Moderate (+) |  |  |  | Food insecurity Lack of access to affordable nutritious food |
| **Community engagement** This activity shares information with the local community on environmental management through household visits. | **Socio-economic** | **29** | 1. Increased education of community members on environmental issues. | 5 | 4 | 1 | 4 | 5 | 17,5 | High (+) | Terrestrial and aquatic habitats, e.g. wetlands, rivers/streams, open space | Presence of natural features of educational value | Environmental education | Poor practices by community members, e.g. littering, dumping, wastage of water |
|  |  | **30** | 2. Increased community cohesion through engagement. | 3 | 4 | 1 | 4 | 4 | 12 | Moderate (+) |  |  |  | Poor communication between community members |
|  |  | **31** | 3. Opportunity to identify and address social issues in the community. | 3 | 3 | 1 | 3 | 2 | 5 | Low (+) |  |  |  | Poor communication between community members |
|  | **Health** | **32** | 4. Reduction in land and water pollution due to improved waste management e.g. reduction in dumping and littering by community members. | 4 | 4 | 2 | 4 | 4 | 14 | Moderate (+) | Terrestrial and aquatic habitats, e.g. wetlands, rivers/streams, open space |  | Water purification | Solid waste pollution Dumping and littering |
| **General Operation of WWWC Programme** | **Socio-economic** | **33** | 1. Improved quality of the life of the beneficiaries and community members. | 5 | 4 | 3 | 5 | 5 | 21,25 | High (+) |  |  |  | Low employment Low education Poverty Lack of access to nutritious food Pollution |
|  |  | **34** | 2. Access to education and training of beneficiaries and potential improved prospects of employment. | 5 | 5 | 3 | 5 | 5 | 22,5 | High (+) |  |  |  | Low education and skills |
|  |  | **35** | 3. New business opportunities for beneficiaries, e.g. SMME. | 3 | 4 | 3 | 4 | 5 | 17,5 | High (+) |  |  |  | Lack of economic opportunities |
|  |  | **36** | 4. Opportunities for co-management of natural spaces between citizens and authorities. | 3 | 4 | 3 | 4 | 4 | 14 | Moderate (+) | Terrestrial and aquatic habitats, e.g. wetlands, rivers/streams, open space | Presence of natural features of educational value | Educational service | Poor service delivery |
|  |  | **37** | 5. Conflict in the community. | 2 | 2 | 1 | 1 | 2 | 3 | Low (-) |  |  |  | Poor communication between community members |

SM Table 2: Intervention Cumulative Impact Scoring

| Nature of impact / outcomes | **Interventions** | | | | | | |  |  |
| --- | --- | --- | --- | --- | --- | --- | --- | --- | --- |
|  | **Solid waste removal** | **Recycling** | **Invasive alien plant removal and control** | **Water quality monitoring** | **Vegetable gardens** | **Community Engagement** | **General** | **Cumulative impact score** |  |
|  |  |  |  |  |  |  |  |  |  |
| **ECOLOGICAL IMPACTS** | **50 (3)** | **7 (1)** | **57 (4)** | **22 (2)** | **0 (0)** | **14 (1)** | **0 (0)** | **150** |  |
| 1.  Improved river water quality | **17,5** |  | **16,25** | **11** |  |  |  | 44,75 |  |
| 2.  Improved river water quantity |  |  | **16,25** |  |  |  |  | 16,25 |  |
| 3.  Improved ecological integrity of terrestrial and aquatic ecosystems | **17,5** |  | **10,5** |  |  |  |  | 28 |  |
| 4.  Reduction in land and water pollution | **15** |  |  | **11** |  | **14** |  | 40 |  |
| 5.  Promotion of indigenous species of flora and fauna. |  |  | **14** |  |  |  |  | 14 |  |
| 6. Reduction in air pollution |  | **7** |  |  |  |  |  | 7 |  |
| **HEALTH IMPACTS** | **16 (3)** | **0 (0)** | **12 (2)** | **7,5 (1)** | **13 (1)** | **0 (0)** | **0 (0)** | **48,5** |  |
| 7.  Reduced safety risks to children and animals | **13** |  | **9** |  |  |  |  | 22 |  |
| 8.  Reduction in diseases linked to pollution, e.g. skin rashes, cholera. | **3** |  |  |  |  |  |  | 3 |  |
| 9.  Health (nutritional) benefits to community through increased accessibility and consumption of a large variety of nutrient rich vegetables. |  |  |  |  | **13** |  |  | 13 |  |
| 10.  Reduction in water related health risks (water borne disease |  |  | **3** | **7,5** |  |  |  | 10,5 |  |
| **SOCIO-ECONOMIC IMPACTS** | **44 (3)** | **38,25 (3)** | **40 (4)** | **9 (1)** | **27 (2)** | **34,5 (3)** | **72,25 (5)** | **265,5** |  |
| 11. Improvement in aesthetic appeal of the area. | **16,25** |  | **7,5** |  |  |  |  | 23,75 |  |
| 12. Increase in recreational and cultural uses of natural areas. | **18,75** |  | **13,75** |  |  |  |  | 32,5 |  |
| 13. Enhancement of agriculture due to improved water quality | **9** |  |  |  |  |  |  | 9 |  |
| 14. Increased income for beneficiaries |  | **13,75** |  |  | **15** |  |  | 28,75 |  |
| 15. Increased environmental education of community |  | **8,25** |  | **9** |  | **17,5** |  | 34,75 |  |
| 16. Reduced load on landfill |  | **16,25** |  |  |  |  |  | 16,25 |  |
| 17. Facilitation of alternative and more appropriate uses of land, e.g. more land available for community gardens. |  |  | **13,75** |  |  |  |  | 13,75 |  |
| 18. Positive economic impact on community members due to availability of cost effective fresh vegetables locally and reduction in need to travel to purchase vegetables |  |  |  |  | **12** |  |  | 12 |  |
| 19. Increased community cohesion |  |  |  |  |  | **12** |  | 12 |  |
| 20. Opportunity to identify and address social issues in the community |  |  |  |  |  | **5** |  | 5 |  |
| 21. Assist government to achieve invasive alien plant control targets |  |  | **5** |  |  |  |  | 5 |  |
| 22. Improved quality of the life of the beneficiaries and community members. |  |  |  |  |  |  | **21,25** | 21,25 |  |
| 23. Access to education and training of beneficiaries and potential improved prospects of employment. |  |  |  |  |  |  | **22,5** | 22,5 |  |
| 24. New business opportunities for beneficiaries, e.g. SMME. |  |  |  |  |  |  | **17,5** | 17,5 |  |
| 25. Opportunities for co-management of natural spaces between citizens and authorities. |  |  |  |  |  |  | **14** | 14 |  |
| 26. Conflict in the community. |  |  |  |  |  |  | **-3** | -3 |  |
| **Cumulative impact score** | **110** | **45,25** | **109** | **38,5** | **40** | **48,5** | **72,25** |  |  |

**SM APPENDIX 2: DESCRIPTIONS FOR INTERVENTIONS IMPACT ASSESSMENT**

***Solid Waste Removal intervention***

The solid waste removal intervention resulted in eight positive outcomes of which three were ecological, two health and three socio-economic (SM Table 1 and SM Table 2). Impacts for the solid waste removal intervention ranged from low to high positive. Improved river water quality improved ecological integrity of terrestrial and aquatic ecosystems and increase in recreational and cultural uses of natural areas scored high positive significance ratings. This intervention also contributed to reduced impact of downstream marine pollution. This activity was linked to the enhancement of ecosystem services, namely, water purification, flood mitigation, waste assimilation, cultural and recreational services. However, the benefits of this intervention were at risk due to continued illegal dumping both by business and trucks coming into the area.

***Recycling intervention***

Four outcomes were identified for the recycling intervention, one of which was ecological and three socio-economic (SM Table 1 and SM Table 2). This intervention provided some income to recyclers and the activity of collecting waste for recycling also educated the broader community on alternative uses of waste. Recycling reduced the load of waste to be collected by service providers and reduced the volume of waste going to the landfill. This recycling activity also reduced the risk of waste being burned, thereby avoiding additional air pollution in the area. The outcomes related to the recycling intervention ranged from low to moderate positive significance, with ‘source of income to recyclers’ and ‘reduced load to landfill’ being ranked as moderate positive.

***Invasive alien plant removal and control***

This intervention resulted in the most outcomes; ten outcomes, four of which were ecological, two health and four were socio-economic impacts (SM Table 1 and SM Table 2). The removal of invasive alien plants through this intervention cleared areas that were previously infested, and opened these areas for alternative uses, e.g., vegetable gardens and recreation (e.g., picnicking), reduced potential for injury and crime and improved the aesthetic appeal of the area. In addition, this activity rehabilitated a wetland and resulted in improved hydrological regulation and the increase of biodiversity in natural areas, e.g., more birds and frogs were noticed by community members. The removal of poisonous invasive alien plants reduced health risks to the community. The impacts related to the removal of invasive alien plants intervention ranged from low to moderate positive. The most significant outcome was ‘improved water quality and quantity,’ which was rated as moderate positive.

***Water quality monitoring***

The water quality monitoring intervention resulted in four outcomes, two of which were ecological, one health and one socio-economic (SM Table 1 and SM Table 2). The monthly measuring of water quality by the beneficiaries facilitated an increased understanding of water quality and pollution levels and assisted to identify sources of pollution. The findings were relayed to the community and thus served as a source of education to community members, which also reduced health risks (e.g., water borne diseases) associated with the use of polluted water. This education resulted in the mitigation of sources of pollution and improved water quality, e.g., increased *E. coli* counts was linked to a location along the river where nappies were dumped, and engagement resulted in nappies no longer being dumped there. The outcomes associated with the monitoring of water quality intervention were of moderate and low positive significance, with ‘improved river water quality and reduction in land and water pollution’ rated as moderate positive in significance.

***Vegetable Gardens***

The vegetable gardening intervention resulted in three outcomes, one of which was health and two were socio-economic (SM Table 1 and SM Table 2). The two vegetable gardens of the programme grew a variety of vegetables, fruit and herbs, which were either consumed by beneficiaries or sold to the local community and local grocery store. The availability of a large variety of vegetables from the WWWC gardens provided an opportunity for beneficiaries and community members to improve their nutritional intake of fresh vegetables, made the produce available in proximity to households and were thus cost effective. The sale of produce by beneficiaries also provided a source of income for them and a means to reinvest into the production of vegetables. Beneficiaries were using natural water for irrigation, which was dependent on water availability that was also threatened by pollution and drought. All the outcomes that resulted from the vegetable gardens intervention were considered to be of moderate positive significance, with the most significant outcome being ‘increased income for beneficiaries through sale of vegetables’.

***Community Engagement***

Eight outcomes were identified from this intervention, five of which were ecological and three socio-economic (SM Table 1 and SM Table 2). The community engagement intervention enhanced social cohesion in the community as it provided for people to feel a sense of inclusion and to build relationships that facilitated the improvement of living conditions and the sharing of knowledge and resources. The regular dialogue with community members provided an opportunity for the community to be educated on environmental matters while gaining an understanding and appreciation of the work the WWWC teams were doing. This education resulted in the improvement of waste and water management practices, and resulted in reduced water wastage and pollution. The outcomes from this intervention ranged from low to high positive significance. The greatest outcome was increased education of community members on environmental issues (high positive significance).

***General operation of the WWWC programmes***

The general operation of the programme resulted in five outcomes, all of which are socio-economic (SM Table 1 and SM Table 2). The quality of life of beneficiaries has improved though the availability of ‘free’ education and training, and the opportunity to gain work experience. This led to the establishment of the first SMME, which was a direct result of the education, training and experience of the beneficiaries through the programme. The establishment of the SMME provided an opportunity for social and economic upliftment of those beneficiaries involved that is both independent and sustainable. The quality of life of the broader community has also been impacted positively, through the WWWC programme, including that the areas are cleaner and safer, there is increased availability and accessibility of fresh vegetables and health benefits. The programme has provided a platform for engagement with authorities and has facilitated the opportunity for the co-management of natural spaces between citizens and authorities (Durban Solid Waste, eThekwini Natural Resources Division and Environmental Planning and Climate Protection Branch).

During the surveys, some issues were raised related to WWWC programme, namely, that some beneficiaries get ‘cost recovery’ and the manner of selection of volunteers to the programme. Although these issues are of low significance due to the fact the people who are part of the programme were already volunteers. The outcomes associated with the general operation of the WWWC programme are mostly high and moderate positive, with only one low negative impact related with minor conflict caused related to some beneficiaries having received cost recovery (provided to beneficiaries for carrying out activities in addition to basic activities), while others did not.
